# Genome sequence of the ectophytic fungus *Ramichloridium luteum* reveals unique evolutionary adaptations to plant surface niche

**DOI:** 10.1186/s12864-017-4118-3

**Published:** 2017-09-15

**Authors:** Bo Wang, Xiaofei Liang, Mark L. Gleason, Rong Zhang, Guangyu Sun

**Affiliations:** 1 State Key Laboratory of Crop Stress Biology in Arid Areas and College of Plant Protection, Northwest A&F University, Yangling, Shaanxi Province 712100 China; 2 Department of Plant Pathology and Microbiology, Iowa State University, Ames, IA 50011 USA

**Keywords:** Sooty blotch and flyspeck, Cutinase, Secretory Lipase, Evolution, Stressful environment

## Abstract

**Background:**

Ectophytic fungi occupy the waxy plant surface, an extreme environment characterized by prolonged desiccation, nutrient limitation, and exposure to solar radiation. The nature of mechanisms that facilitate adaptation to this environment remains unclear. In this study, we sequenced the complete genome of an ectophytic fungus, *Ramichloridium luteum*, which colonizes the surface of apple fruit, and carried out comparative genomic and transcriptome analysis.

**Results:**

The *R. luteum* genome was 28.18 Mb and encoded 9466 genes containing 1.85% repetitive elements. Compared with cell-penetrating pathogens, genes encoding plant cell wall degrading enzymes (PCWDEs), PTH11-like G protein-coupled receptors (GPCRs) and effectors were drastically reduced. In contrast, genes encoding cutinases and secretory lipases were strikingly expanded, and four of nine secretory lipases were probably acquired by horizontal gene transfer from Basidiomycota. Transcriptomic analysis revealed elevated expression of genes involved in cuticle degradation (cutinase, secretory lipase) and stress responses (melanin biosynthesis, aquaporins, lysozymes and HOG pathway).

**Conclusions:**

Taken together, our results highlight genomic features associated with evolution of surface niche adaptation by the ectophytic fungus *R. luteum*, namely the contraction of PCWDEs, PTH11-like GPCRs and effectors, and the expansion of cuticle degradation and stress tolerance.

**Electronic supplementary material:**

The online version of this article (10.1186/s12864-017-4118-3) contains supplementary material, which is available to authorized users.

## Background

Plant-colonizing microorganisms occupy two broad types of ecological niche: interior and surface [1]. Invasive phytopathogens, symbionts and endophytes establish populations in the intracellular or apoplastic spaces, which constitute the interior niche [2, 3]. Epiphytic and ectophytic organisms, on the other hand, colonize primarily the surface niche. Ectophytes differ from epiphytes in that they interact with and absorb nutrients from extracellular components of plant surfaces [4–6].

Spanu [2] concluded that ancestral saprophytic fungi evolved into cell-penetrating plant pathogens under suitable conditions. Adaptations required for invasive plant pathogenesis include expansion and diversification of plant cell wall depolymerases, effectors and metabolic phytotoxins [2]. In contrast, sooty blotch and flyspeck (SBFS) fungi, which descended from invasive plant pathogens, colonize epicuticular layers of plants as ectophytes [6]. They can extract required nutrients from living hosts without killing cells and tissues. During the evolutionary transition from invasive plant pathogenesis to an ectophytic niche [6, 7], most pathogenicity-related genes except some associated with cuticle penetration were lost in SBFS fungus *Peltaster fructicola* [6]*.* Transition from invasive pathogenesis to the ectophytic niche represents an important but little-studied pathway in plant-fungus co-evolution.

SBFS can cause substantial economic damage worldwide on fruit of apple, pear, persimmon and many other cultivated crops by forming superficial dark-colored blemishes which downgrade fruit for fresh-market sale and accelerate fruit desiccation during cold storage [8]. SBFS fungi include more than 80 species, most of which belong to the order Capnodiales within the class Dothideomycetes [8]. SBFS species are polyphyletic, exhibiting convergent evolution of their specialized ectophytic colonization lifestyle [9–14].

The plant surface micro-environment is characterized by direct sunlight exposure, a hydrophobic waxy surface, and scarcity of available water and nutrients. To survive, ectophytic fungi must evolve strategies to deal with these abiotic stresses. In addition, ectophytes must also compete with other surface-dwelling microorganisms for nutrient absorption and evade or counteract the toxic effects of plant-derived antimicrobial metabolites [15]. Although living as an ectophyte could reduce elicitation of host countermeasures, ectophytes must cope with the harsh environment at the plant surface. During surface colonization, SBFS fungi produce darkly pigmented (melanized) hyphae, which can enable them to resist extreme temperatures, desiccation, and ionizing radiation [16]. Xu et al. [6] sequenced the genome of the SBFS fungus *P. fructicola* and found that genes controlling 1,8-dihydroxynaphthalene (DHN)-melanin biosynthesis are highly expressed on the apple surface. However, the full range of adaptations by SBFS fungi to plant surfaces remains unclear. Moreover, it is unclear whether such genomic features are general characteristics of the SBFS fungal complex.

In this study, we carried out genomic and transcriptomic sequencing of a SBFS fungus in the genus *Ramichloridium*, *R. luteum* G.Y. Sun, H.Y. Li & Crous [13]. The genus *Ramichloridium* Stahel ex de Hoog accommodates a wide range of species with diverse lifestyles [13, 17]. Batzer et al. [9] provided evidence that *Ramichloridium* is in Chaetothyriales whereas *Peltaster* is in Dothideales, indicating that *Ramichloridium* spp. differ from *P. fructicola* at the level of taxonomic order. Morphologically, species in genus *Ramichloridium* also differ notably from those in *Peltaster* [17, 18]. The main goals of this work were to understand genomic features underlying the evolutionary adaptation of *R. luteum* to the plant surface niche and to compare adaptions for ectophytic colonization to those of *P. fructicola*.

## Results

### General genome features

The final genome assembly of *R. luteum* contained 155 scaffolds with a total length of 28.18 Mb and a coverage of 161-fold. *R. luteum* genome size was considerably smaller than the average genome size of Ascomycota (36.91 Mb) [19], but over 55% larger than the SBFS fungus *P. fructicola* (genome size 18.14 Mb) [6]. The genome scaffold N50 was 437 kb, the largest scaffold was 1.7 Mb, and there were 108 scaffolds longer than 3 kb. Seven assembly scaffolds ended with the typical fungal telomere repeat (TTAGGG)_n_. The completeness of the genome assembly was assessed by the CEGMA, which gave 93.95% full coverage and 95.56% partial coverage. The GC content of the genome was 54.72% and the genome contained 22 rRNAs and 78 tRNAs (Additional file 1: Table S1).

The repeat content of *R. luteum* was 1.85% as determined by RepeatModeler and RepeatMasker with very low repetitive content of transposable elements (TEs, 0.8%). The repeat sequence profile indicated a significant repeat-induced point mutation (RIP) in the *R. luteum* genome, where the TpA/ApT ratio was 1.20 and the (CpA + TpG)/(ApC + GpT) ratio was 0.83. The *R. luteum* genome contained the DNA methyltransferase RID gene required for RIP.

The *R. luteum* genome encoded 9466 predicted proteins, among which 5816 (61.44%), 5857 (61.87%), 3301 (34.87%), and 8775 (92.70%) could be annotated based on Gene ontology (GO) (Additional file 2: Figure S1), Clusters of Orthologous Groups (COG) (Additional file 3: Figure S2), Kyoto Encyclopaedia of Genes and Genomes (KEGG), and NCBI nr databases, respectively.

### Phylogenomic analysis and protein family evolution

**Fig. 1:**
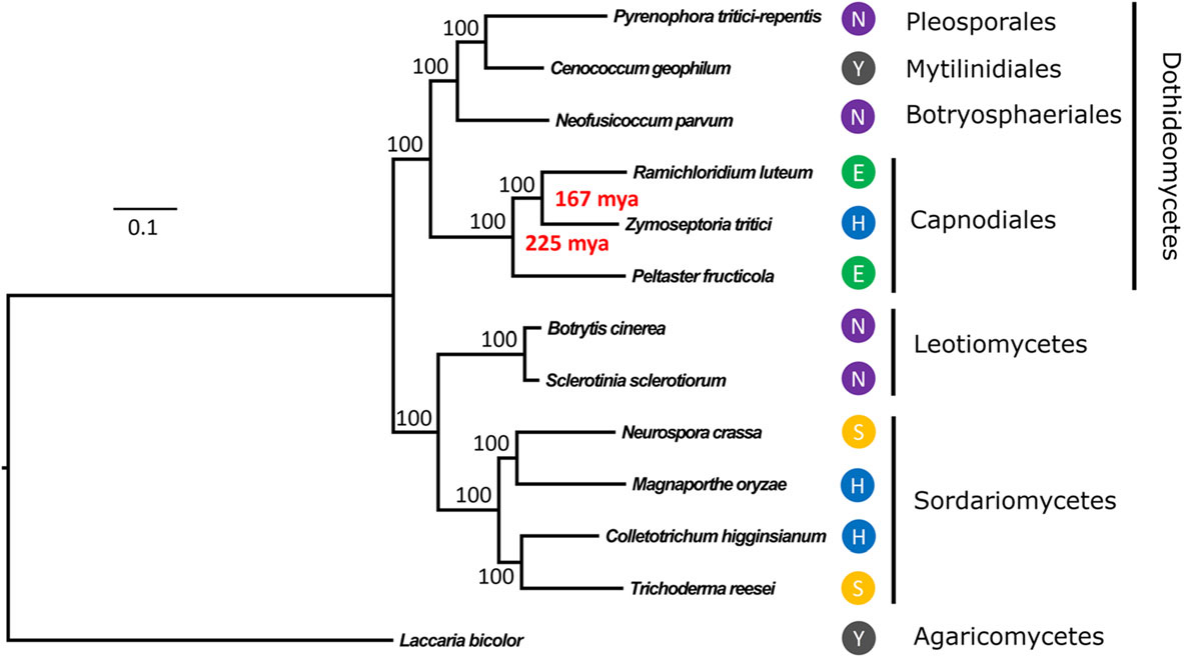
A maximum likelihood phylogenetic tree constructed from concatenated alignment of 1656 single-copy orthologs conserved across all 13 species using RAxML with the best-fit model of LG + I + G + F. Bootstrap values based on 100 replicates are shown for indicated nodes. Red color represents the divergence time in million years. Boxes represent the life style of selected organisms. E, ectophytes; H, hemibiotrophs; N, necrotrophs; S, saprotrophs; Y, symbionts

**Fig. 2:**
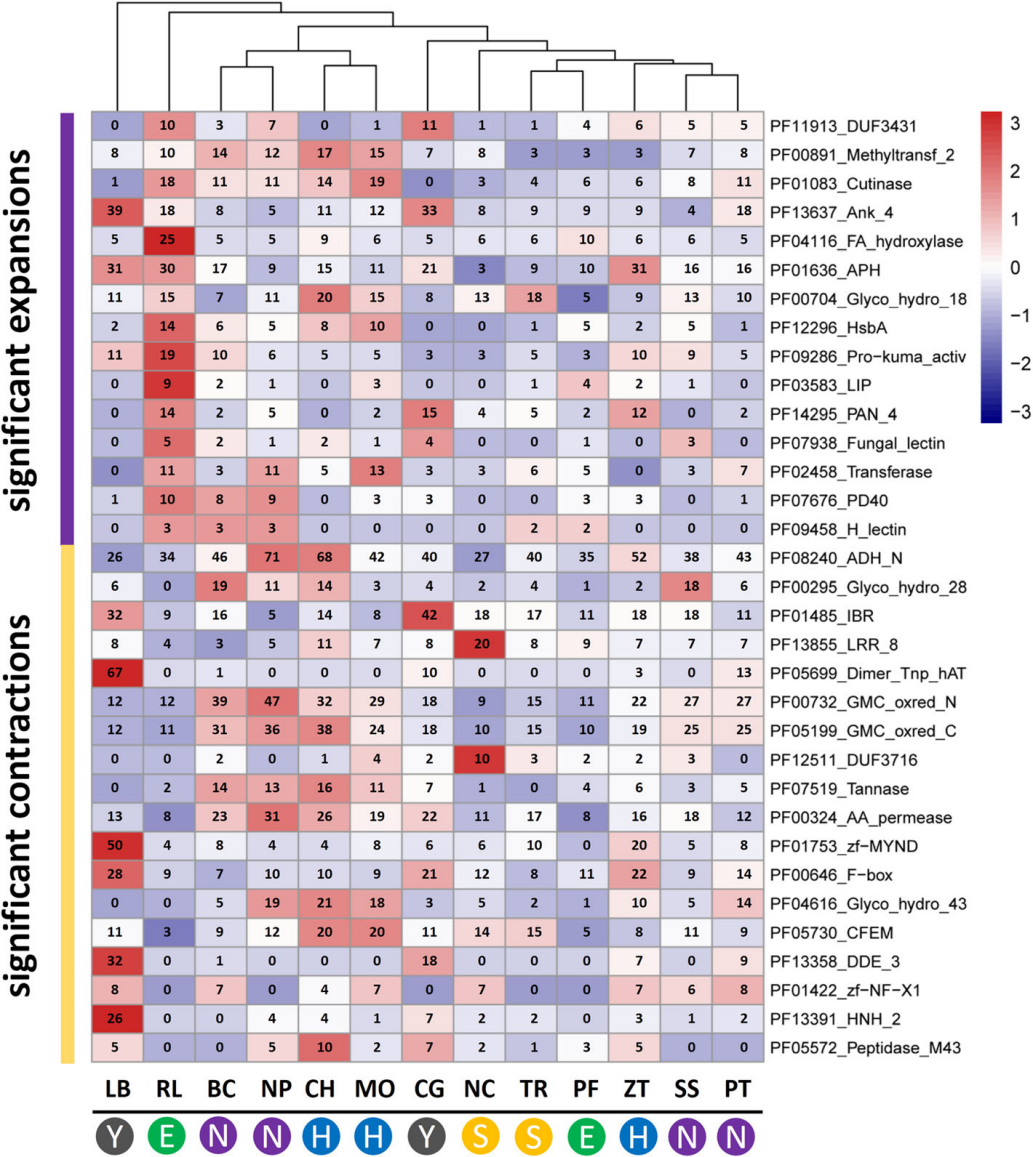
Significant expansions and contractions of protein family in *R. luteum*. Evolution analysis was processed by CAFE. Numbers of protein family members in each genome are shown. Fungal names: BC = *Botrytis cinerea*; CH = *Colletotrichum higginsianum*; LB = *Laccaria bicolor*; MO = *Magnaporthe oryzae*; ZT = *Zymoseptoria tritici*; NP = *Neofusicoccum parvum*; NC = *Neurospora crassa*; PF = *Peltaster fructicola*; PT = *Pyrenophora tritici-repentis*; RL = *Ramichloridium luteum*; SS = *Sclerotinia sclerotiorum*; TR = *Trichoderma reesei*; CG = *Cenococcum geophilum*. Overrepresented (+3 to 0) and underrepresented (0 to −3) numbers are depicted as Z-scores for each line in heatmap. The boxes on the nethermost represent the life style of the selected organisms. E, ectophytes; H, hemibiotrophs; N, necrotrophs; S, saprotrophs; Y, symbionts

Genomes of 13 species of fungi with various lifestyles including symbionts, free-living saprophytes and invasive phytopathogens were selected for comparison. A total of 1656 single-copy orthologs conserved across all compared species (Additional file 1: Table S2) were selected to construct the phylogenomic tree. All tree branches received 100% bootstrap support, indicating a highly reliable topological structure (Fig. 1). The results showed that the *Ramichloridium* lineage was evolutionarily closer to the penetrating plant pathogen *Zymoseptoria tritici* than to the SBFS species *P. fructicola*. The r8s analysis showed that the divergence time between *R. luteum* and *Z. tritici* was around 167 million years ago (mya), whereas that between *R. luteum* and *P. fructicola* was around 225 mya. This phylogenetic pattern indicates that the ectophytic lifestyles of *R. luteum* and *P. fructicola* have evolved independently. CAFE analysis showed that protein families including cutinase, secretory lipase, fatty acid hydroxylase, and hydrophobic surface binding protein A (HsbA) were expanded and protein families including PWCDEs, proteins containing cysteine-rich domain (CFEM domain), LRR_8 domain and zf-MYND domain involved in protein-protein interactions were contracted in *R. luteum* (Fig. 2).

### Contraction of PTH11-like GPCRs and candidate effectors

The PTH11 G protein-coupled receptors (GPCRs) are a family of cell surface integral membrane proteins required for pathogenicity [20, 21]. Proteins in this family contain seven transmembrane regions and an amino-terminal extracellular CFEM domain. The number of predicted CFEM proteins varied from 3 in *R. luteum* to 20 in *Magnaporthe oryzae* or *Colletotrichum higginsianum* (Fig. 2). *R. luteum* had fewer CFEM proteins than *P. fructicola* (5 members) and *Z. tritici* (8 members)*.* CAFE analysis confirmed the CFEM contraction in *R. luteum* (*P* = 0.003). The three *R. luteum* proteins contained 0–1 transmembrane helices, ruling out the possibility that they are PTH11-like GPCRs.

**Fig. 3:**
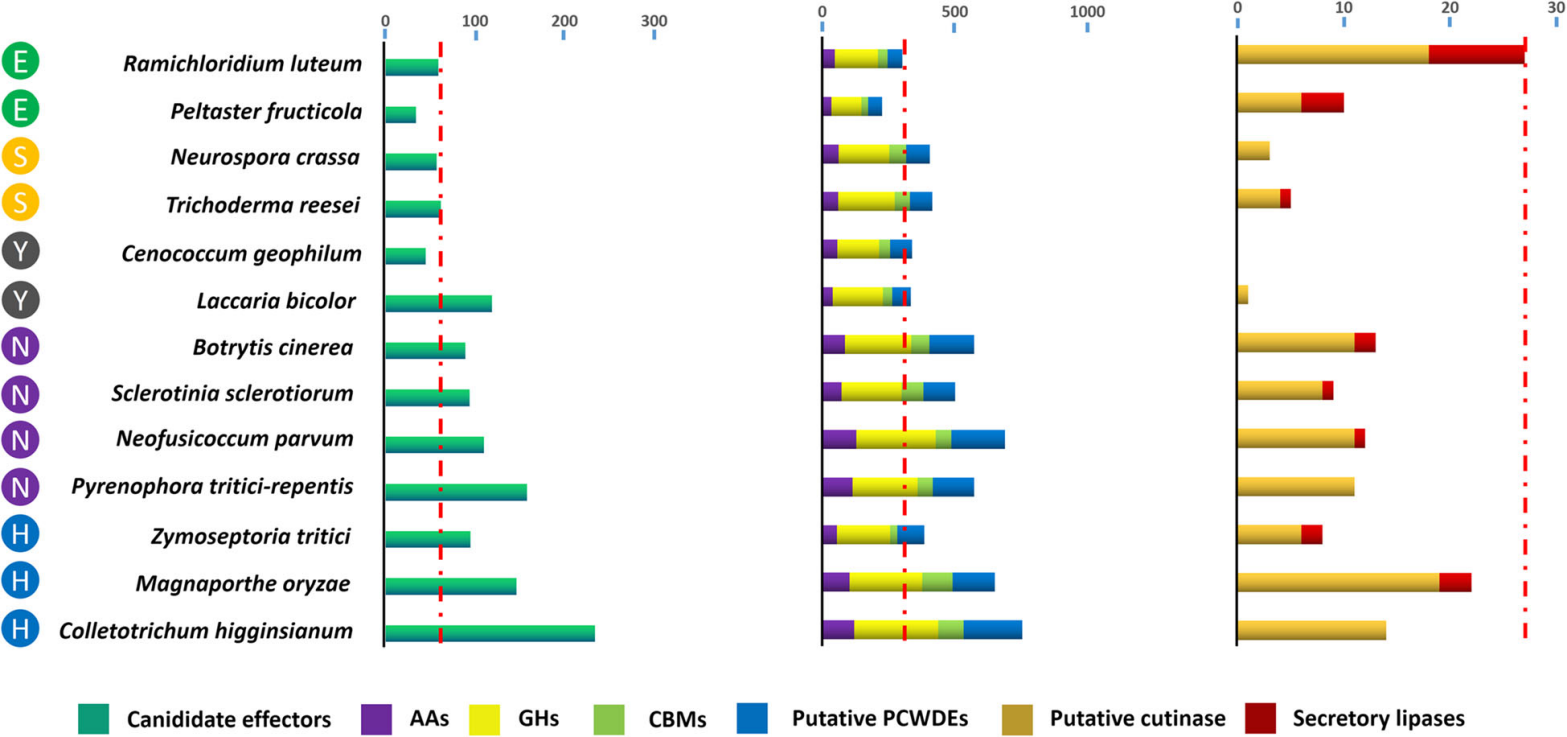
Numbers of genes encoding putative candidate effectors, CAZY modules (AAs, auxiliary activities; GHs, glycoside hydrolases; CBMs, carbohydrate-binding modules), PCWDEs, putative cutinases and secretory lipases identified in the genomes of *R. luteum* and 12 additional fungal species included in this study. Boxes on the left represent the life style of the selected organisms. E, ectophytes; H, hemibiotrophs; N, necrotrophs; S, saprotrophs; Y, symbionts

Effector proteins of plant pathogenic fungi are low-molecular-weight, typically cysteine-rich, secreted compounds that are key virulence factors modulating host defense reactions and cellular activities to facilitate infection [22]. Effector is also necessary for the mutualistic symbionts facilitating hyphal entry into the root and required for microbial-microbial growth competitions [23–25]. Candidate effectors (putatively secreted, cysteine-rich, <200 aa) ranged from 34 in the ectophytic fungus *P. fructicola* to 234 in the cell-penetrating species *C. higginsianum* (Fig. 3). *R. luteum* had 59 such proteins, more than the penetrating symbiont Dothidiomycete *Cenococcum geophilum* (45), similar to the saprotrophic fungi *Neurospora crassa* (57) and *Trichoderma reesei* (62), but significantly fewer than penetrating plant pathogens (ranging from 89 to 234) and the penetrating symbiont Basidiomycota *Laccaria bicolor* (119). The hemibiotrophic plant pathogen Z. *tritici* had 95 candidate effectors, more than both *R. luteum* and *P. fructicola* but fewer than all host-penetrating species except for the necrotrophic fungus *Botrytis cinerea* (89). Overall, *R. luteum* has a significantly reduced effector gene content compared with penetrating phytopathogens.

Fortynine of the 59 candidate effectors in *R. luteum* contained unknown domains, whereas only 10 contained a PFAM domain, indicating an enrichment of genes with putatively unknown function (Additional file 1: Table S3). Eight genes among 59 candidate effectors were up-regulated during ectophytic growth. One of 8 up-regulated genes, Ramle7664 is taxon-specific since it is found only in *R. luteum*. The up-regulation of expression of putative effectors is conceivably required for adaptation to a surface niche, such as warding off microbial antagonists.

### Loss of plant cell wall-degrading enzymes

**Fig. 4:**
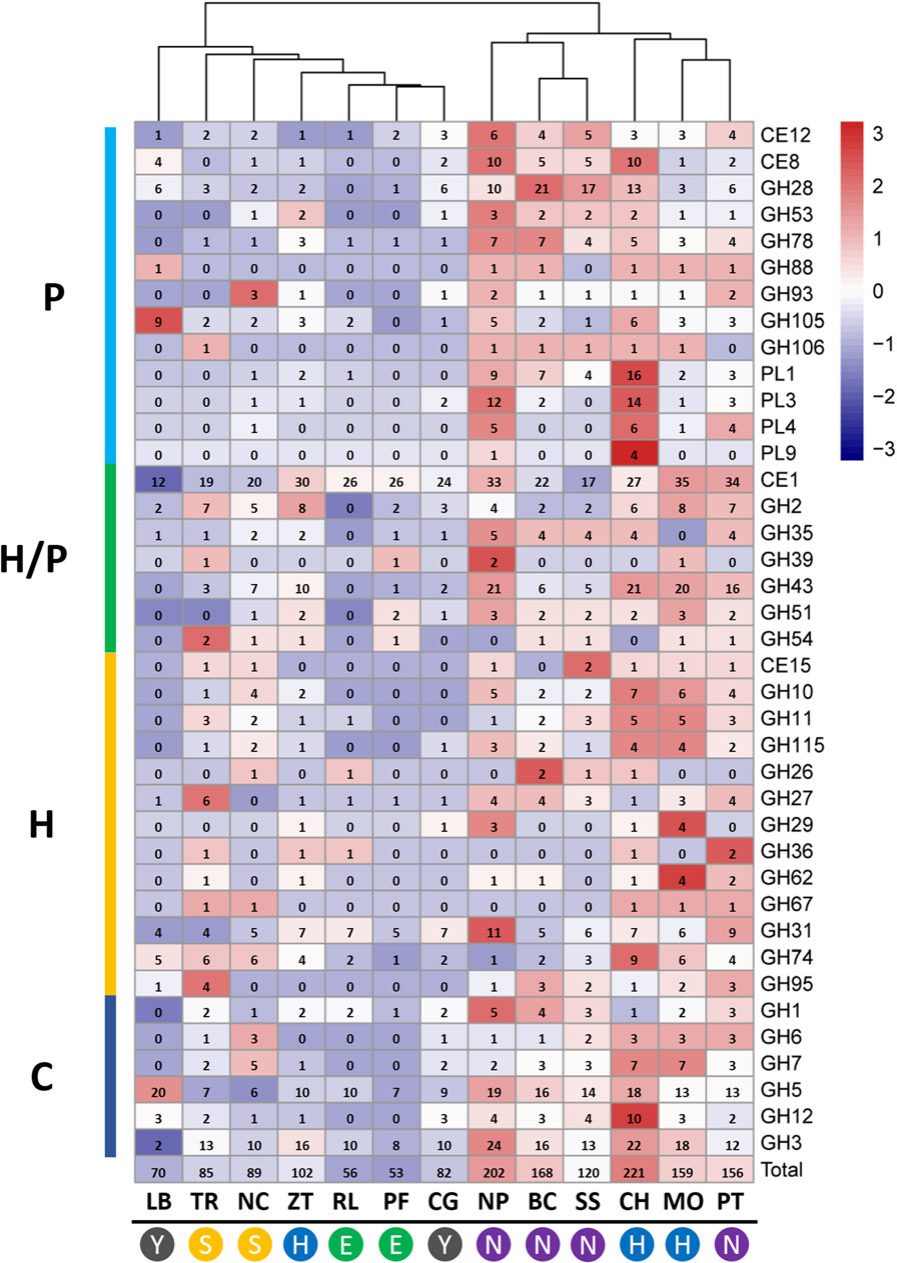
Heatmap showing the distribution of putative plant cell wall degrading enzymes among different fungi. Numbers of family members in each genome are shown. Fungal names: BC = *Botrytis cinerea*; CH = *Colletotrichum higginsianum*; LB = *Laccaria bicolor*; MO = *Magnaporthe oryzae*; ZT = *Zymoseptoria tritici*; NP = *Neofusicoccum parvum*; NC = *Neurospora crassa*; PF = *Peltaster fructicola*; PT = *Pyrenophora tritici-repentis*; RL = *Ramichloridium luteum*; SS = *Sclerotinia sclerotiorum*; TR = *Trichoderma reesei*; CG = *Cenococcum geophilum*. Overrepresented (+3 to 0) and underrepresented (0 to −3) numbers are depicted as Z-scores for each line in heatmap. Boxes on the nethermost represent the life style of the selected organisms. E, ectophytes; H, hemibiotrophs; N, necrotrophs; S, saprotrophs; Y, symbionts. Boxes on the right represent the CAZY substrates. P, pectin; H, hemicellulose; C, cellulose

The *R. luteum* genome contained 474 carbohydrate active enzyme (CAZY) modules (Additional file 1: Table S4), which was more than *P. fructicola* (363), *L. bicolor* (427) and *C. geophilum* (436), but fewer than other plant pathogenic and saprophytic fungal species (493 to 839). Furthermore, CAZY annotation demonstrated that *R. luteum* contains a much smaller set of glycoside hydrolases (GHs) (163 vs. average 218, **, *P* < 0.005, *t*-test), auxiliary activities (AAs) (47 vs. average 75, **, *P* = 0.008, *t*-test), and carbohydrate binding modules (CBMs) (36 vs. average 58, *, *P* = 0.011, *t*-test) compared to the average number for all 13 species (Fig. 3). The strong reduction of GHs, AAs and CBMs in *R. luteum* suggested a reduced capacity to degrade plant and fungal cell walls. Plant cell walls are composed mostly of cellulose, hemicellulose and pectin, and pathogens attack them by secreting PCWDEs [26]. We further found that *R. luteum* and *P. fructicola* had similar numbers of PCWDEs, 56 and 53 respectively, from CAZY genes, which were much fewer than *Z. tritici* (102 members) and other all non-SBFS fungal species with various lifestyles (**, *P* < 0.01, *t*-test) (Fig. 3). Numbers of PCWDE genes in SBFS fungi were fewer than in the penetrating symbiont fungi *L. bicolor* and *C. geophilum* (70 and 82, respectively). Hierarchical clustering analysis showed that the PCWDE profile of *R. luteum* is most closely related to that of *P. fructicola* and *C. geophilum*, followed by *Z. tritici* [27] (Fig. 4)*.* CAFE analysis showed that GH28 (0 vs. average 7.2) and GH43 (0 vs. 8.4) were most strikingly reduced in *R. luteum* (*P* < 0.05), and there was one GH28 and one GH43 in *P. fructicola* (Fig. 2), indicating that putative enzymes involved in the degradation of pectin were contracted in SBFS fungi. Only 5 genes involved in pectin degradation in *R. luteum* and 4 genes in *P. fructicola* were found (Fig. 4). These numbers are less than for penetrating symbiont fungi (17 to 21) and penetrating phytopathogens (16 to 82). Fungal enzymes involved in pectin backbone degradation mainly included pectate lyases belonging to PL1, PL3, PL4, and PL9 families and hydrolases belonging to GH family 28 [28]. *R. luteum* lacked all GH28 genes for enzymes that degrade pectin backbones, such as endopolygalacturonase (PGA), exopolygalacturonase (PGX), endorhamnogalacturonase (RHG), exorhamnogalacturonase (RGX). Moreover, *R. luteum* had only one pectin lyase, Ramle7389 (PEL: PL1), which was expressed similarly *in planta* and in vitro. *R. luteum* grew more slowly on a medium containing apple pectin as the sole nutrient source than that containing glucose; this result correlated with a reduced repertoire for pectin degradation (Additional file 5: Figure S4). The ectophytic fungi *P. fructicola* had one enzyme (GH28) involved in pectin lyase backbones and *Z. tritici* had five genes (two GH28, two PL1 and one PL3), which was fewer than for symbiotic fungi (av. = 7) and penetrating plant pathogens (av. = 25) (Fig. 4).

*R. luteum* and *P. fructicola* were also reduced in regard to β-1,4-xylosidases (BXL: GH3, GH43) that release D-xyloses from non-reducing ends of xylooligosaccharides (Fig. 4). The numbers of GH3 and GH43 in *R. luteum* and *P. fructicola* were much lower than in *Z. tritici* or the average of cell-penetrating plant pathogens (10 and 9 vs. 26 and 31, respectively). Cellulose is a polymer of β-1,4-linked D-glucose residues, the degradation of which is mediated by three classes of enzymes in synergy: β-1,4-endoglucanases (EGL: GH5, 7 and 12), exoglucanases/cellobiohydrolases (CBH: GH6 and GH7), and β-glucosidase (BGL: GH1 and GH3) [28]. Cellulase genes of *R. luteum, P. fructicola, Z. tritici* and the average of penetrating plant pathogens numbered 22, 16, 30 and 62 respectively. Interestingly, *R. luteum* and *P. fructicola* processed only EGL and BGL, whereas *Z. tritici* processed all three classes.

Collectively, reduction in PCWDEs content appeared to be an ancient trait for the phylogenetic clade containing *R. luteum*, *P. fructicola*, and *Z. tritici*. However, the fact that *R. luteum* and *P. fructicola* contained many fewer PCWDEs than *Z. tritici* suggested a secondary round of PCWDE gene reduction associated with adaptation to an ectophytic niche. In addition, RNA-seq data showed that only two secreted PCWDE-coding genes, Ramle6973 and Ramle1916 (GH3, for releasing of terminal glucoses from shorter oligosaccharides), were moderately expressed and up-regulated *in planta* (Additional file 1: Table S5).

### Expansion of cutinase and secretory lipase

**Fig. 5:**
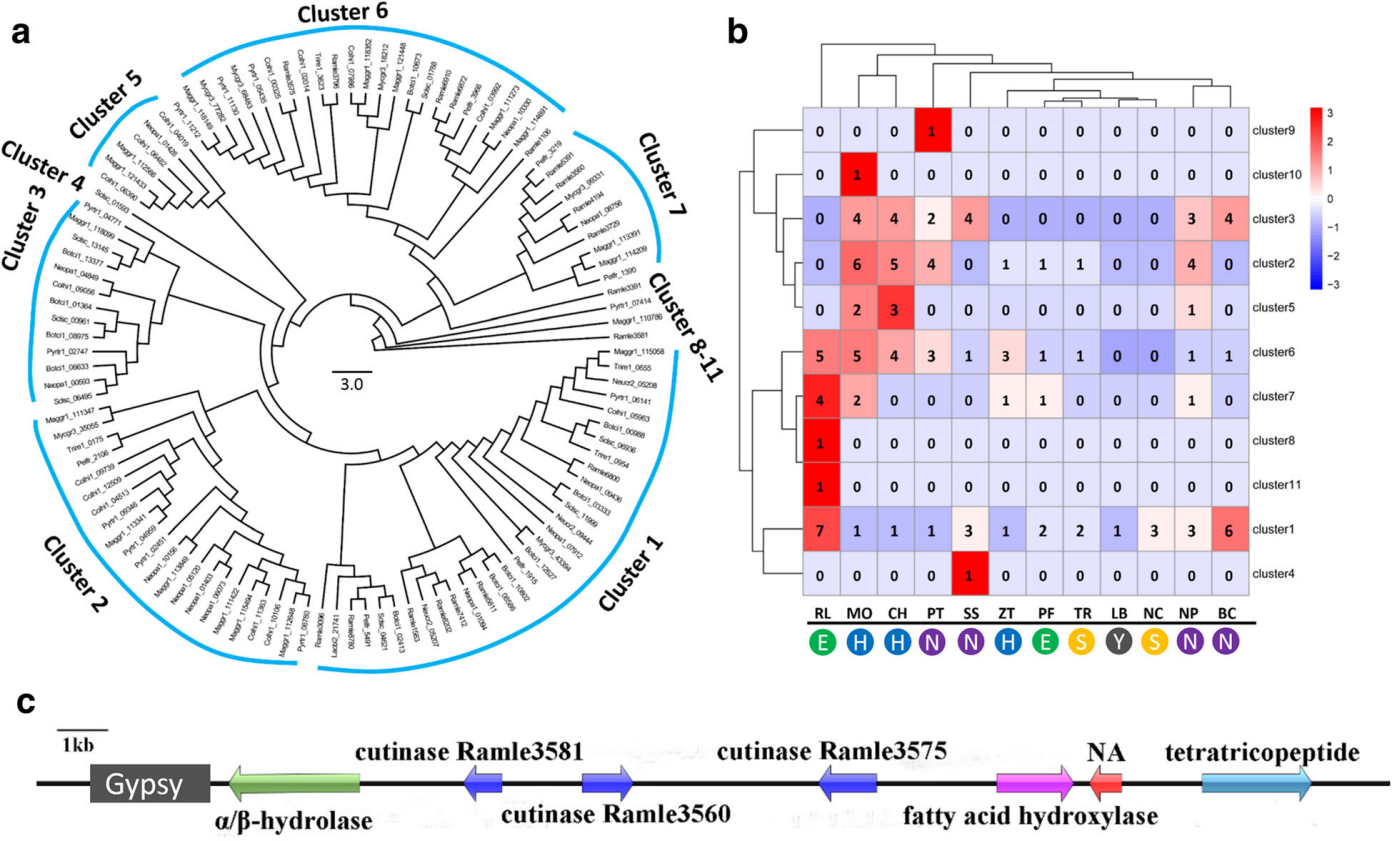
Striking expansion of putative cutinase genes in *R. luteum*. **a** phylogenetic tree of predicted cutinases among the 13 compared fungal species. **b** gene copy number variation among the phylogenetic clusters defined in A. Numbers of putative cutinase genes in each genome are shown. Overrepresented (+3 to 0) and underrepresented (0 to −3) numbers are depicted as Z-scores in heatmap. **c** local tandem duplication of cutinase genes in *R. luteum.* Botci1 = *Botrytis cinerea*; Maggr1 = *Magnaporthe oryzae*; Mycgr3 = *Zymoseptoria tritici*; Neopa1 = *Neofusicoccum parvum*; Pelfr = *Peltaster fructicola*; Ramle = *Ramichloridium luteum*; Sclsc = *Sclerotinia sclerotiorum*; Trire1 = *Trichoderma reesei*; Lacbi2 = *Laccaria bicolor*; Pyrtr1 = *Pyrenophora tritici-repentis*; Colhi1 = *Colletotrichum higginsianum*; Neucr2 = *Neurospora crassa*. E, ectophytes; H, hemibiotrophs; N, necrotrophs; S, saprotrophs; Y, symbionts

Cutinase and secretory lipase are key enzymes degrading plant cutin and wax derivatives, and are presumably important for surface niche adaptations [29–31]. CAFE analysis showed that both categories of enzymes were significantly expanded in the *R. luteum* genome (*P* < 0.05) (Fig. 2). Of a total of 13 fungal species surveyed in this work, *R. luteum*, together with the rice blast fungus *M. oryzae*, encoded the highest number of putative cutinases (PF01083, 18 members), which was far more than the average number (9). The difference was even more striking in the Dothideomycetes, in which the previously highest documented number was 14 in *Cochliobolus sativus* and *C. heterostrophus* (http://pfam.xfam.org). Interestingly, this increase in cutinase copy number was not observed with the SBFS fungus *P. fructicola* (6) or in the non-SBFS *Z. tritici* (6). Phylogenetic analysis identified 11 clusters out of 112 putative cutinases (Fig. 5a). *R. luteum* cutinases were distributed among clusters 1, 6, 7, 8 and 11. Gene expansion was noted in clusters 1, 6 and 7 (Fig. 5b). Clusters 8 and 11 contained two species-specific cutinase genes, Ramle3391 and Ramle3581. Interestingly, cutinases Ramle3581 (cluster 11), Ramle3560 (cluster 7) and Ramle3575 (cluster 6) form a tandem repeat cluster (Fig. 5c), indicating local duplication events over the course of cutinase evolution in this species. Meanwhile, this cluster was surrounded by genes encoding α/β-hydrolase and fatty acid hydroxylase, and there was also a Gypsy retroelement (~2 kb in length) in the 5′-flanking region of Ramle3581 (<4 kb).

HsbA is a secreted protein which facilitates the binding between cutinase and hydrophobic surfaces, thus facilitating cutin degradation [32]. The genome of *R. luteum* contained significantly more HsbA than penetrating plant pathogen species (14 vs. 1–7) (Fig. 2), and 13 of these proteins were predicted to be secreted.

**Fig. 6:**
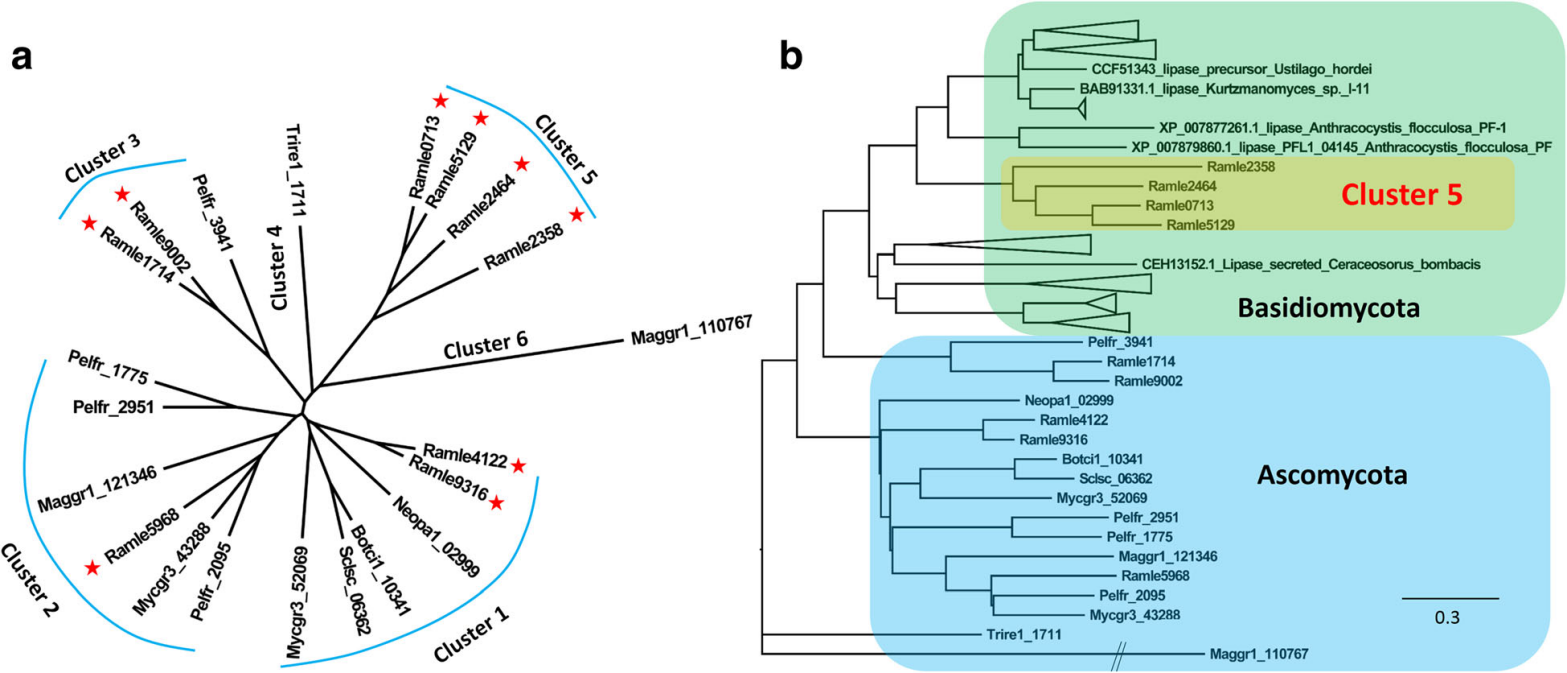
Expansion of secretory lipases in *R. luteum* and their horizontal transfer from Basidiomycota. **a** phylogenetic tree of secretory lipases among the 13 compared fungal species. **b** phylogenetic tree constructed with *R. luteum* proteins in cluster 5 together with their best BLASTp hits in NCBI nr database. Botci1 = *Botrytis cinerea*; Maggr1 = *Magnaporthe oryzae*; Mycgr3 = *Zymoseptoria tritici*; Neopa1 = *Neofusicoccum parvum*; Pelfr = *Peltaster fructicola*; Ramle = *Ramichloridium luteum*; Sclsc = *Sclerotinia sclerotiorum*; Trire1 = *Trichoderma reesei*

The *R. luteum* genome encoded nine secreted lipases (domain PF03583), which is exceeds the number in *P. fructicola* and *Z. tritici* (4 and 2, respectively) and is significantly higher than the average number found in all fungal species in this study (2, ***, *P* < 0.001, *t*-test,). Phylogenetically, lipases from the 13 compared species were divided into six clusters (Fig. 6a), with clusters 3 and 5 being specific to the SBFS fungi, *R. luteum* and *P. fructicola*. Interestingly, genes in cluster 5 were most similar to Basidiomycota sequences based on a BLASTp search (Additional file 1: Table S6). Ramle0713 was selected for illustration. When the protein was searched against the NCBI nr database with BLASTp, 46 hits (more than 200 alignment scores), all belonging to Basidiomycota, were applicable for building phylogenies. The results of phylogenetic analysis, shown in Fig. 6b, strongly suggest that cluster 5 genes have been transferred from Basidiomycota through horizontal gene transfer.

**Fig. 7:**
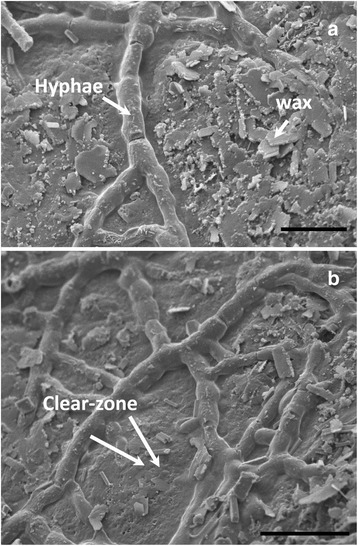
*R. luteum* could efficiently degrade apple cuticles by scanning electron microscopy (SEM) observation. (**a**) SEM microscopy showing the disappearance of waxy crystals around hyphal network. (**b**) SEM microscopy showing the formation of a clearing zone surrounding ectophytic hyphae. Scale bars: a = 10 μm; b = 20 μm

Based on RNA-seq data, two secretory lipases (Ramle1714 and Ramle9316) and one cutinase (Ramle6800) were highly expressed and up-regulated at least two-fold *in planta*. In addition, one HsbA homolog (Ramle4244) was significantly up-regulated by ~3-fold at the colonization stage. During ectophytic colonization of the apple fruit surface, we observed an apparent disappearance of waxy crystals around the hyphae network (Fig. 7a). Later, clearing zones devoid of waxy crystals started to form within the hyphae network (Fig. 7b). The microscopic observations suggested that *R. luteum* could degrade apple cuticle efficiently.

### Fungal surface adaptation and stress responses

Microorganisms on surface niches are directly exposed to atmospheric extremes and sunlight. Furthermore, the hydrophobic waxy cuticle that covers plant epidermal cells reduces evaporation of water inside the fruit and the leaching of substances from plants [33, 34], thus resulting in an oligotrophic environment [35]. Reproduction on the plant surface requires adaptation to this habitat, including the ability to withstand abiotic and biotic stresses such as scarce nutrients and water, UV irradiation, and antagonism by other microorganisms. The following transcriptomic analyses focus on expression of traits of *R. luteum* in its ectophytic niche.

**Fig. 8:**
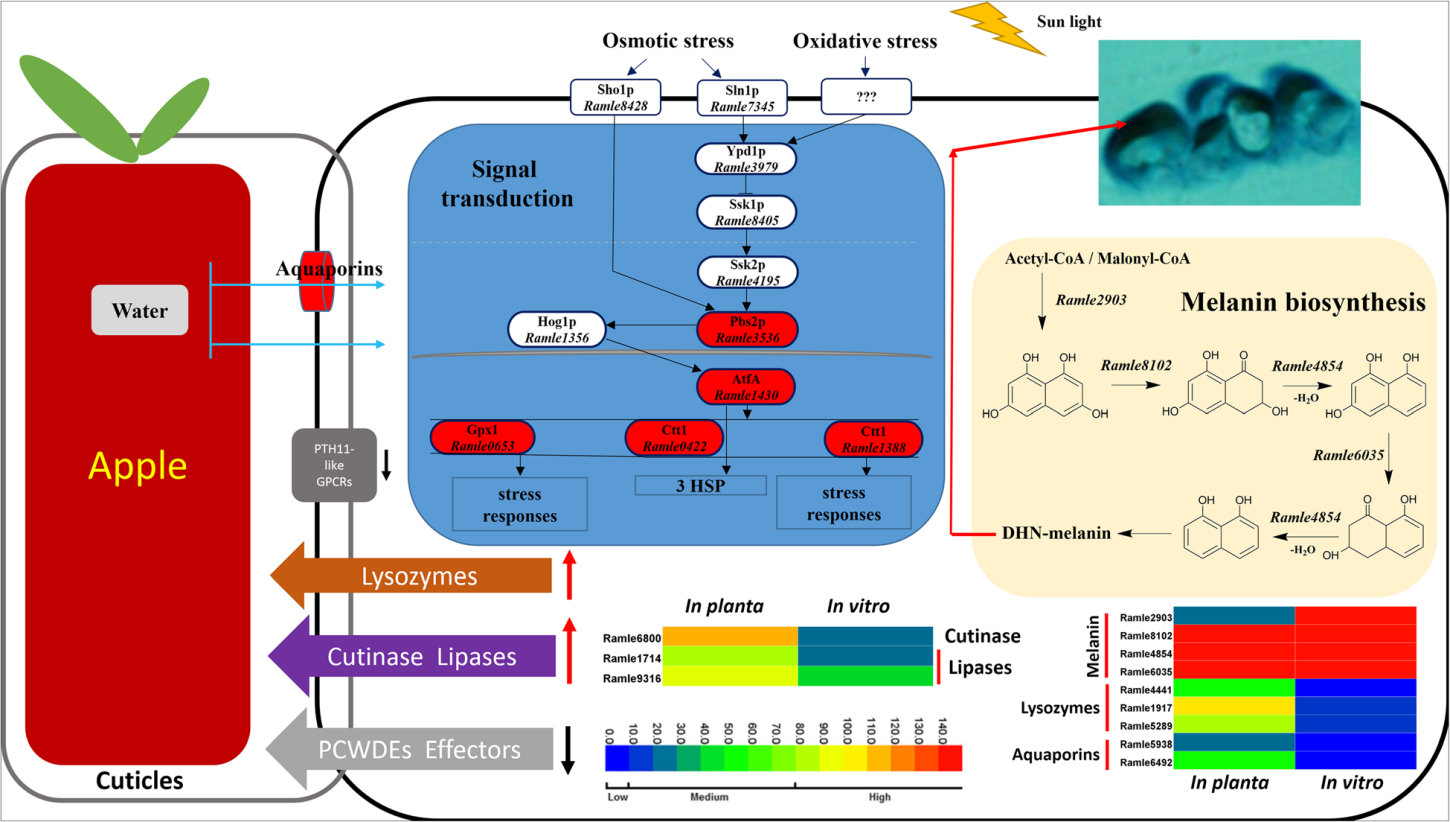
Proposed surface niche adaptation model for the ectophytic SBFS fungus *R. luteum*. Plant cell wall degrading enzymes (PCWDEs), PTH11-like G protein-coupled receptors (GPCRs) and effectors were drastically reduced. In contrast, genes encoding cutinases, secretory lipases and GH25-lysozymes were strikingly expanded. Genes involved in cuticle degradation (cutinase, secretory lipase) and stress responses (melanin biosynthesis, aquaporins, lysozymes and HOG pathway) were activated by *R. luteum* colonizing in its ectophytic niche. For heatmaps, two columns represent different treatments, i.e., inoculation on apple fruit (*in planta*) and growth on artificial media (in vitro), and each row is marked with the name of one gene. The colored scale bar of expression levels is divided into three grades: low (0 < FPKM <10, including 0), medium (10 < FPKM <80), and high (80 < FPKM)

**a)** ***Melanin*** Melanin can protect fungi from UV irradiation, desiccation, high temperatures, and strong oxidants [36]. On fruit surfaces, *R. luteum* hyphae exposed to air are heavily melanized. In most Ascomycota species, melanin is synthesized via the 1,8-DHN biosynthesis pathway [36]. The *R. luteum* genome encodes a complete 1,8-DHN biosynthetic pathway, which includes PKS1 (Ramle2903), 1,3,6,8-tetrahydroxynaphthalene reductase (Ramle8102), scytalone dehydratase (Ramle4854), and 1,3,8-trihydroxynaphthalene reductase (Ramle6035). RNA-Seq data indicated that these genes were expressed similarly *in planta* and in vitro, with PKS1 expressed at an intermediate level whereas the other three genes were expressed at a high level (Fig. 8).

**b)** ***Lysozyme*** The CAZY GH25 family exhibits lysozyme-mediated lytic activity, which attacks peptidoglycans in the cell walls of several bacterial species [37]. Interestingly, the *R. luteum* genome encoded three GH25 proteins whereas other fungal genomes encode one at most. All three lysozymes (Ramle1917, Ramle4441 and Ramle5289) were up-regulated *in planta*, increasing 8-, 15- and 6-fold, respectively (Fig. 8). These putative lysozymes may give the *R. luteum* a competitive advantage over bacteria on the fruit surface.

**c)** ***Aquaporin*** Aquaporins are integral membrane proteins which form water-transporting pores on membrane surface and play critical roles in osmotic stress responses [38]. The *R. luteum* genome encoded three aquaporin-like proteins, two of which (Ramle5938 and Ramle6492) were up-regulated *in planta*, with the induction fold being 3 and 25 respectively (Fig. 8).

**d)** ***High osmolarity glycerol (HOG) pathway.*** The HOG pathway is an important mitogen-activated protein kinase (MAPK) signaling pathway that is able to detect, transduce signals and respond to osmotic and oxidative stresses [39, 40]. The HOG MAPK pathway was complete in the *R. luteum* genome (Additional file 1: Table S7). The MAPK kinase Pbs2p (Ramle3536) and the downstream bZip transcription factor AtfA (Ramle1430) were up-regulated by approximately two- and three-fold, respectively, *in planta* compared to in vitro. Several putative AtfA downstream targets were strongly up-regulated *in planta*; examples include three heat shock proteins (Ramle2917, Ramle1564 and Ramle5908, exhibiting fold increases of 718, 74, and 37, respectively), antioxidative genes like glutathione peroxidase (Ramle0653, 2-fold increase), and two catalases (Ramle0422 and Ramle1388, fold increases of 143 and 37, respectively) (Fig. 8).

The above results suggested that melanin, the HOG pathway and aquaporin play key roles in protecting ectophytic fungi from UV irradiation, desiccation, high temperatures and oxidants, and that putative lysozymes may help them to compete with other microorganisms. For adaptation to an oligotrophic environment, greatly expanded cutinase and lipase production could increase the efficiency of degradation of the hydrophobic waxy cuticle to enhance access to sugar-rich fruit leachates.

## Discussion

The plant cuticle is an extracellular lipophilic biopolymer made up mainly of cutin and epicuticular wax [41, 42]. Cutinase hydrolyzes primarily cutin whereas lipase acts mainly on epicuticular waxes [29]. Our comparative genomic analysis revealed that cutinase and secretory lipase are strikingly expanded in the *R. luteum* genome, which is in accordance with our microscopic observation that this fungus can actively degrade the cuticle during apple surface colonization. The *R. luteum* genome contains three cutinases forming tandem duplications, which occur near (<4 kb) the repeat retrotransposon Gypsy, indicating that local gene duplication contributes to gene family expansion. Furthermore, based on our phylogenetic analysis *R. luteum* may have acquired secretory lipases from Basidiomycota fungi via horizontal gene transfer.

In contrast with *R. luteum*, histological studies of the SBFS fungi *P. fructicola*, *Leptodontidium elatius*, *Zygophiala jamaicensis* and *Z. wisconsinensis* suggested that these fungi do not efficiently degrade the wax and cuticle layers during colonization [6, 43–45]. It is therefore possible that different SBFS fungi have evolved different capacities for degrading plant cuticles despite the fact that they are all well-adapted to the surface niche. Moreover, the *R. luteum* and *P. fructicola* genomes differ strikingly in the number of putative cutinases (18 vs. 6) and secretory lipases (9 vs. 4).

On the other hand, despite their long evolutionary separation (over 200 mya), the *R. luteum* and *P. fructicola* genomes share many signatures. For example, both genomes were drastically reduced in size, repeat elements, genes involved in plant cell wall degradation, PTH11-like GPCRs and effectors in comparison to host cell-penetrating species. The substantial divergence in phylogeny, together with similarity in genome reduction, indicate that their genome reductions are the result of convergent evolution. The most closely related penetrating phytopathogenic species, *Z. tritici*, also showed reduction in these features when compared with other fungi. Thus, the reductions are phylogenetically related and likely are results of contractions from the genomes their most recent common ancestor. On the other hand, even when compared with *Z. tritici*, the reduction of PCWDEs, effectors, and PTH11-like GPCRs was still substantial in both *R. luteum* and *P. fructicola*. This suggests that there was a subsequent round of virulence-related gene content reduction in the two SBFS fungi that was associated with niche adaptation.

A reduced complement of PCWDEs is meaningful for pathogenic and mutualistic interactions of biotrophs and hemibiotrophs to avoid triggering plant defense mechanisms and establish a biotrophic lifestyle [2, 46]. Ectomycorrhizal fungi colonizing the extracellular spaces also benefit from PCWDEs to penetrate the roots, although they exhibited a general reduction of such genes [46, 47]. Interestingly, numbers of PCWDEs in *R. luteum* and *P. fructicola* were fewer than for the Dothidiomycete species *C. geophilum* and even the symbiont Basidiomycete *L. bicolor*. Even when compared to other ectomycorrhizal fungi in Agaricomycetes [48], the number of CAZY associated with plant cell wall degradation are fewer in the SBFS species. This result may indicate evolutionary adaptations to the ectophytic lifestyle; i.e., colonizing epicuticular layers of plants and extracting nutrients from the surface of living hosts without penetrating and killing cells.

Our data also highlight the importance of stress response genes for ectophytic colonization by *R. luteum*. On the plant surface, microorganisms must deal with ultraviolet radiation and low or fluctuating water availability. Moreover, microorganisms must compete with other surface-inhabiting organisms. In bacteria, protection mechanisms that were shown experimentally to be important in epiphytic fitness involve the production of pigments and the activation of DNA repair mechanisms, catalases and superoxide dismutases [35, 49–51]. In the present study, we found a high level of expression of genes involved in melanin biosynthesis, aquaporins, heat shock proteins, catalases and peroxidase processing by *R. luteum.* Interestingly, *R. luteum* expanded three CH25-lysozymes during its evolution, which is more than any of the other 12 fungal species we used for comparison, suggesting a unique mechanism to deal with biotic stress at the plant surface.

## Conclusion

In this study, we reported the genome sequence of *Ramichloridium luteum*, one of the representative SBFS species on apple in China [13]. Comparing the genome and transcriptome of this ectophytic species with *P. fructicola*, a first model of surface colonization, as well as those of fungi that occupy other ecological niches, has yielded new insight into the evolutionary adaptability and life strategy of ectophytic fungi. *R. luteum* and *P. fructicola* share many genomic similarities related to surface niche adaptation. For instance, compared with penetrating plant-pathogenic species and penetrating symbiont fungi, both genomes showed a dramatic reduction in pathogenicity-related genes. Our data supports the ectophytic phytopathogen evolutionary model proposed by Xu et al. [6], a pathway from interior to exterior colonizers. In fact, colonization by stealth is strategic for an ectophyte in order to reduce the risk of triggering defensive countermeasures by the host. Moreover, both SBFS fungi share similarity in genes controlling 1,8-DHN-melanin biosynthesis, which were expressed at a high level on the plant surface. On the other hand, *R. luteum* has strikingly more genes encoding cutinase and secretory lipase than *P. fructicola*, suggesting a scenario whereby different SBFS fungi have evolved distinct strategies to ensure successful surface niche adaptation. Generating and analyzing additional SBFS genomes will be necessary for discerning common genome features associated with their surface niche adaptations.

## Methods

### Fungal strain and culture conditions

Strain CPC 18961 of *R. luteum* was originally isolated from the surface of an apple fruit showing SBFS symptoms near Weifang City, Shandong Province, China. The culture was single-spore purified, maintained on potato dextrose agar (PDA) medium at 25°C, and stored in glycerol (15%) at -80°C in the Fungal Laboratory of Northwest A&F University, Yangling, Shaanxi Province, China.

### DNA isolation, genome sequencing and assembly

Highly purified total genomic DNA was isolated from the fungal mycelia collected from a 2-week old PDA culture following the modified cetyltrimethyl ammonium bromide protocol [52]. The genome of *R. luteum* was sequenced with the Illumina HiSeq™ 2000 platform. The insertion size of the sequencing library was 360 bp and the sequencing strategy was 100 bp pair-ends. Filtered clean reads were assembled into scaffolds using the ABySS assembler v. 1.3.5 [53]. The completeness of assembly was assessed using CEGMA v. 2.4 [54]. GapFiller software was used to further fill the gaps and generate scaffolds [55].

### Gene prediction and genome annotation

GeneMark-ET was first used to predict gene structures based on RNA-seq data [56]. Obtained gene models were used to train Augustus v. 3.1 [57]. Predicted gene models from GeneMark-ET and Augustus, as well as the homology proteins of *Capnodiales*, were combined in MAKER2 [58]. InterProScan and Pfam were used to identify protein domains [59, 60]. Protein encoding genes were annotated through BLASTp search against the GO [61], COG [62], KEGG [63], and non-redundant NCBI databases, at the threshold of *E*-value ≤1 × 10^−5^. Repeat sequences were identified by RepeatMasker v. 4.0.5 (http://www.repeatmasker.org) and RepeatModeler v. 1.0.7 [64], and non-coding RNA was predicted by rRNAmmer v. 1.2 (http://www.cbs.dtu.dk/services/RNAmmer), tRNAscan-SE v. 1.3.1 [65] and Rfam (http://rfam.xfam.org). For calculation of RIP indices, dinucleotide frequencies were determined using the RIPCAL program [66].

### Functional annotation of predicted genes

Genes encoding putative CAZY were identified using the hmmscan program by searching the fungal proteomes with the family-specific HMM profiles of CAZY downloaded from the dbCAN database [67]. CAZY families corresponding to PCWDEs were in accordance with previous research [68]. Melanin synthase genes were identified by searching with *Cochliobolus heterostrophus* homologs [69]. Stress-responsive proteins were identified by BLASTp search with queries from other fungal species such as *Saccharomyces cerevisiae*, *S. pombe* and *Aspergillus nidulans* [40].

Candidate secreted proteins have a secretion signal as determined by SignalP v. 4.1 [70] and have no transmembrane domain as determined by TMHMM 2.0 (http://www.cbs.dtu.dk/services/TMHMM), and finally having extracellular score > 15 as predicted by WoLF-PSort v. 0.2 software [71]. Small secreted proteins are defined here as proteins that are smaller than 200 aa and were labeled as ‘cysteine rich’ when the percentage of cysteine residue was at least twice as high as the average percentage in entire proteomes [72].

### Phylogenomic analysis

OrthoMCL v. 2.0.9 was used to identify ortholog pairs among compared genomes. The cutoff *E*-value was set as 1 × 10^−5^ [73]. To construct a genome-based phylogenic tree, single-copy ortholog pairs were aligned with MAFFT v. 7 (http://mafft.cbrc.jp/alignment/server), conserved sites in the alignments were further extracted with Gblocks v. 0.91b using the default parameters [74], and the dataset was used for maximum likelihood tree construction in RAxML [75] with the LG + I + G + F amino acid substitution model selected by ProtTest v. 3.4 [76]. Divergence times between species were estimated using the PL method with r8s [77]. The estimated Sordariomycota divergence (290–380 million years ago) was used for calibration [78]. MEGA v. 7.0 was used to generate maximum likelihood phylogeny for cutinase and secretory lipase with the JTT (Jones, Taylor, and Thorton) amino acid substitution model [79, 80]. Statistical support for phylogenetic grouping was assessed by 1000 bootstrap re-samplings. The program CAFE (Computational Analysis of gene Family Evolution) v. 3 [81] was used to analyze gene family size evolution, and the Viterbi algorithm in the CAFE program was used to identify families going through significant expansions/contractions at each branch using a cutoff of *P* < 0.05.

### Inoculation experiment

The *R. luteum* isolate CPC 18961 was used to inoculate apple fruit in the field following the described protocol [9]. Prior to inoculation, single-conidium-derived isolates were cultured on PDA for 1 month. Five mycelial colonies were harvested, excessive agar was cut away, colonies were transferred to five 1.5-ml plastic centrifuge tubes (one colony per tube) to which 600 μl sterile deionized water (SDW) was added, and the mixture was shaken on a vortex oscillator (Model QL-901, Kylin-Bell Lab Instruments Company Limited, Haimen City, Jiangsu Province, China) for 60 s. After that, they were refrigerated until use, which was within 2 h of preparation [45].

Immature apple fruit (cv. Fuji; 5–6 cm in diameter) were selected for in situ inoculation. Fruit surfaces were sterilized by swabbing with 70% ethanol, air-dried, and then swabbed with inoculum suspension using sterilized brushes. In total, 10 fruit were inoculated and 10 fruit were treated only with SDW as a control. All 20 fruits were placed in sealed transparent polyethylene bags (20 × 15 cm) (The CLOROX Company, Oakland, California); two corners (1.5 × 1.5 cm) of each bag were cut off each bag to facilitate aeration. After approximately 3 months of incubation (temperature range from 11^o^ C to 37°C), apples with visible colonies of *R. luteum* were harvested for total RNA extraction and microscopic examination.

### Transcriptome analysis

Mycelia from artificial inoculations onto apple fruit in the field were used as ‘*in plant*a’ samples for RNA sequencing. For ‘in vitro’ mycelium, *R. luteum* was cultured in liquid Czapek-Dox medium [82] for 7 d at 25°C with a shake speed of 150 rpm. For ‘*in planta*’ and ‘in vitro’ treatments, three independent biological samples were collected for RNA extraction and sequencing. The average cDNA library was 350 bp. The libraries were paired-end sequenced on a flow cell using an Illumina HiSeq™ 2500 sequencing platform with a read length of 125 bp. After filtration, reads were mapped to the genome sequence using TopHat v. 2.0.9 with the parameter “-g 1” [83]. Reads mapped to multiple locations were discarded. In total, 29,060,627 RNA-seq reads were mapped to the *R. luteum* genome (Additional file 1: Table S8). A gene expression profile was created using Cufflinks v. 2.2.1 [84]. FPKM (fragments per kilobase of transcript per million fragments mapped) values were used to determine Pearson’s correlation coefficient among biological replicates (0.84 to 0.99) (Additional file 4: Figure S3a). We performed principal component analysis (PCA) to assess biological variability among all samples (Additional file 4: Figure S3b); FPKM of transcripts with a significant *P*-value (<0.05) and a greater than twofold change (log2) in transcript level were considered to be differently expressed. All *P*-values were corrected for false discoveries resulting from multiple hypothesis testing using the Benjamini-Hochberg procedure.

### Scanning electron microscopy

Epicarps with sooty blotch colonies were peeled off the fruit and cut into 5 mm × 5 mm pieces, then air dried for 48 h. Dehydrated samples were mounted on stubs, sputter coated with gold-palladium [85] and viewed using a HITACHI S-3400N scanning electron microscopy operating at 5 KV.

### Apple pectin utilization

For determining physiological correlates of gene reduction associated with pectin degradation, we grew the SBFS fungi on media containing pectin as a sole source of carbon. We used water-agar (Bacto™ Agar, USA) medium as the basal medium, amended with 0.2% apple pectin (Sigma-Aldrich, USA) as a sole carbon source and 0.2% glucose as a sole carbon source (Guangdong Guanghua Sci-Tech Co. Ltd., China), respectively. For strain growth, 1 μl of conidial suspension (concentration: 1 × 10^−6^ CFU/ml) was inoculated onto each plate; the culture was incubated at 25°C for 10 d and then photographed. Water-agar medium was set as the internal control medium. There were six plates per treatment.

## Additional files

Additional file 1: Table S1. Genome characteristics of *R. luteum.* **Table S2.** Species used in this comparative study. **Table S3.** The expression of 59 candidate effectors in *R. luteum*. Green fills represent the up-regulated genes on ectophytic growth. **Table S4.** Number of carbohydrate-active enzyme modules of *R. luteum* and 12 other fungi according to the CAZY database. GHs = Glycosyl hydrolases; GTs = Glycosyl transferases; CBMs = Carbohydrate-binding modules; CEs = Carbohydrate esterase; AAs = Auxiliary activities; PLs = Polysaccharide lyases. **Table S5.** Up-regulated genes involved in PCWDEs of *R. luteum* on the apple surface. **Table S6.** Nine secretory lipases show best-hit relationships with the fungi in NCBI nr database. **Table S7.** Orthologues and putative orthologues in response regulator-dependent osmotic and oxidative stress-response regulatory in *R. luteum.* **Table S8.** General features of data got by the high throughput transcriptome analysis. (XLS 72 kb)

Additional file 2: Figure S1. GO functional classification of the *R. luteum* genome. In total, there are 5816 genes (61.44%) that have functional assignments. (TIFF 1334 kb)

Additional file 3: Figure S2. Distribution of COG function annotation of the *R. luteum* genome. In total, there are 5857 genes (61.87%) that have functional assignments. (TIFF 700 kb)

Additional file 4: Figure S3. Relationships of biological samples in RNA-seq experiment. (a) Pearson’s correlation outcomes; (b) PCA plot. (TIFF 543 kb)

Additional file 5: Figure S4. Comparative growth profiling of *R. luteum* on two carbon substrates. (a) On water-agar medium; (b) On water-agar medium containing apple pectin as a sole carbon source; (c) On water-agar medium containing glucose as a sole carbon source. (TIFF 5055 kb)

## Abbreviations

1,8-DHN: 1,8-dihydroxynaphthalene
BLAST: Basic Local Alignment Search Tool
CAFE: Computational Analysis of gene Family Evolution
COG: Clusters of Orthologous Groups
FPKM: Fragments Per Kilobase of transcript per Million fragments mapped
GPCRs: G Protein-Coupled Receptors
HOG: High Osmolarity Glycerol
HsbA: Hydrophobic surface binding protein A
KEGG: Kyoto Encyclopaedia of Genes and Genomes
MAPK: Mitogen-Activated Protein Kinase
MEGA: Molecular Evolutionary Genetic Analysis
PCWDEs: Plant Cell Wall Degrading Enzymes
RIP: Repeat-Induced Point mutation
SBFS: Sooty Blotch and FlySpeck
SEM: Scanning Electron Microscopy
