# Supplementary figures and images for: Genome sequence of the ectophytic fungus Ramichloridium luteum reveals unique evolutionary adaptations to plant surface niche
Source: BMC Genomics. 2017 Sep 15;18:729. doi: 10.1186/s12864-017-4118-3 (PMC5602860; doi:10.1186/s12864-017-4118-3)

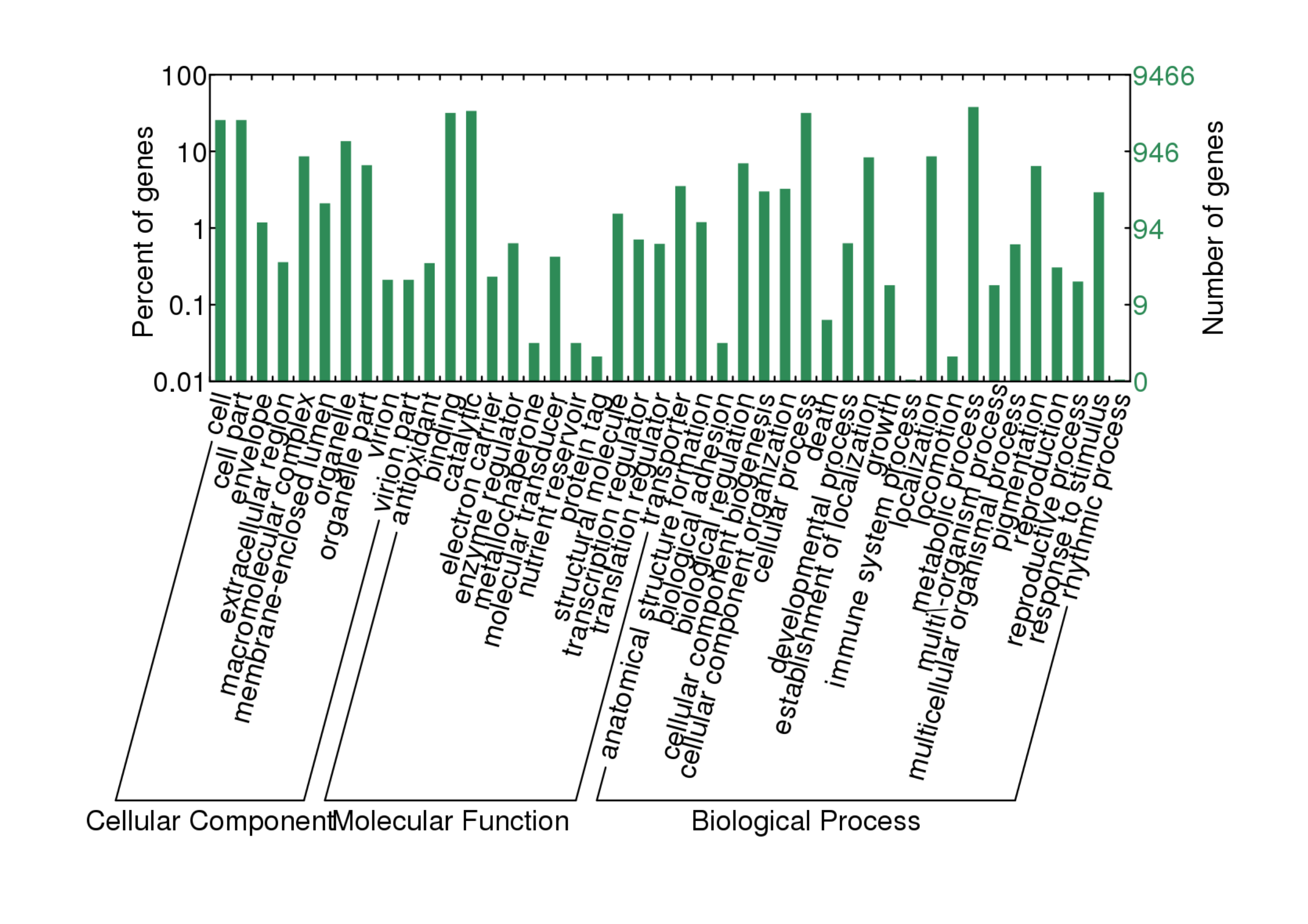

Supplement: Supplementary file 2 — GO functional classification of the R. luteum genome. In total, there are 5816 genes (61.44%) that have functional assignments. (TIFF 1334 kb) [file 12864_2017_4118_MOESM2_ESM.tif]

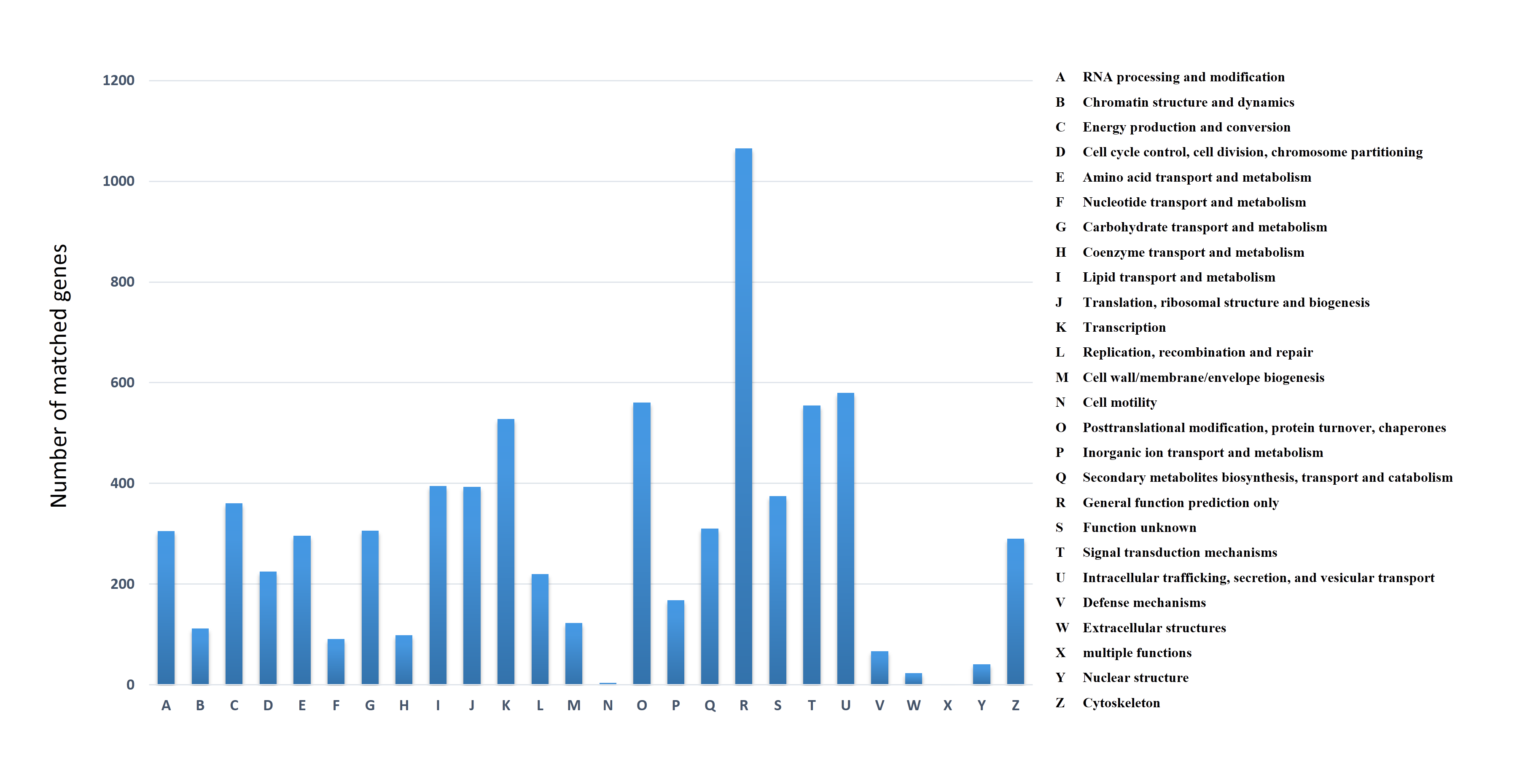

Supplement: Supplementary file 3 — Distribution of COG function annotation of the R. luteum genome. In total, there are 5857 genes (61.87%) that have functional assignments. (TIFF 700 kb) [file 12864_2017_4118_MOESM3_ESM.tif]

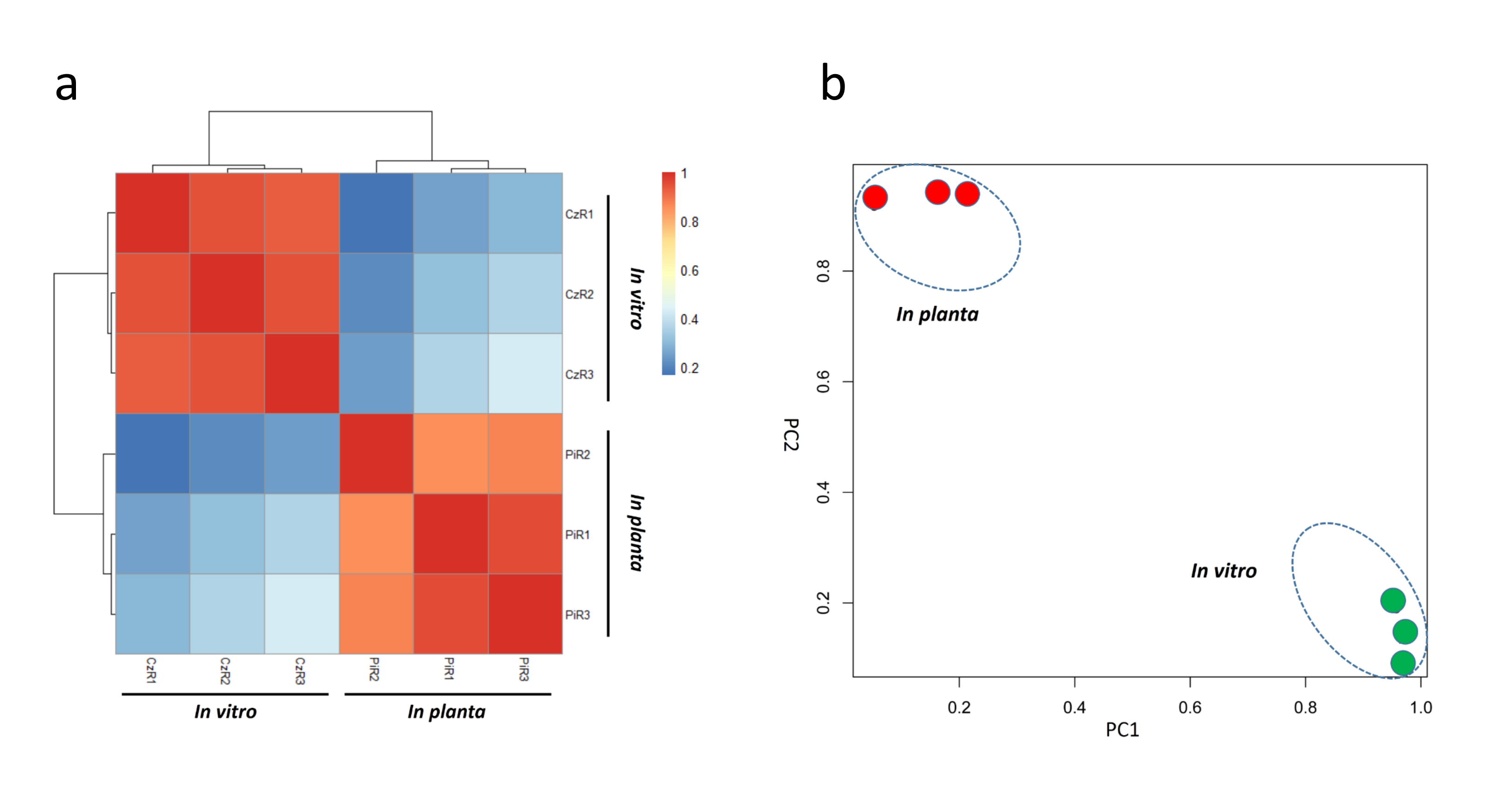

Supplement: Supplementary file 4 — Relationships of biological samples in RNA-seq experiment. (a) Pearson’s correlation outcomes; (b) PCA plot. (TIFF 543 kb) [file 12864_2017_4118_MOESM4_ESM.tif]

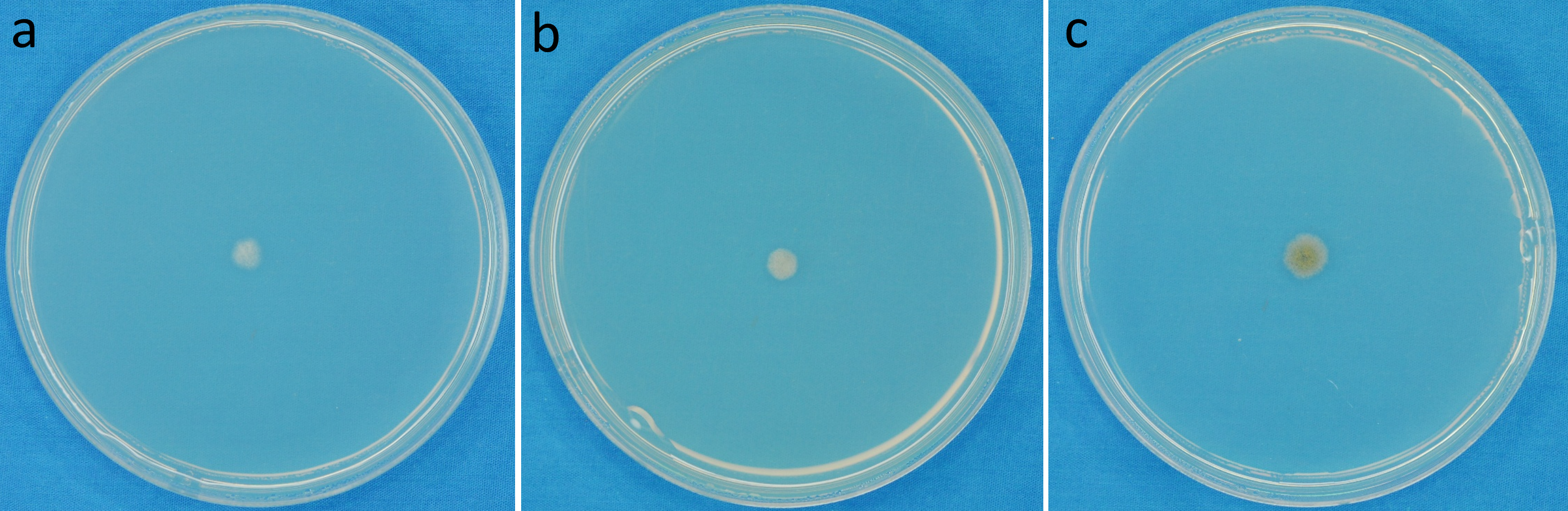

Supplement: Supplementary file 5 — Comparative growth profiling of R. luteum on two carbon substrates. (a) On water-agar medium; (b) On water-agar medium containing apple pectin as a sole carbon source; (c) On water-agar medium containing glucose as a sole carbon source. (TIFF 5055 kb) [file 12864_2017_4118_MOESM5_ESM.tif]
